# Emergence and characterization of IncFII/IncR plasmids with multiple 5,692 bp- *bla*_KPC−2_-bearing tandem repeats in ceftazidime/avibactam non-susceptible *Klebsiella pneumoniae* strains

**DOI:** 10.3389/fmicb.2025.1534631

**Published:** 2025-04-03

**Authors:** Hongmao Liu, Mei Zhu, Junwan Lu, Shan Wu, Rujian Ye, Wei Pan, Yirong Li, Qiyu Bao, Dawei Huang

**Affiliations:** ^1^Department of Laboratory Medicine, Zhongnan Hospital of Wuhan University, Wuhan, China; ^2^School of Laboratory Medicine and Life Sciences, Institue of Biomedical Informatics, Wenzhou Medical University, Wenzhou, China; ^3^Department of Clinical Laboratory, Zhejiang Hospital, Hangzhou, China; ^4^Medical Molecular Biology Laboratory, School of Medicine, Jinhua University of Vocational Technology, Jinhua, China; ^5^Department of Clinical Laboratory, The People's Hospital of Yuhuan, Taizhou, China

**Keywords:** *Klebsiella pneumoniae*, Ceftazidime/avibactam, blaKPC-2, hypervirulent, 5,692 bp-tandem repeat

## Abstract

Ceftazidime/avibactam (CAZ/AVI) is widely recognized as an effective treatment for infections caused by KPC-producing *Klebsiella pneumoniae* (KPC-Kp). However, the prevalence of CAZ/AVI resistance among KPC-Kp isolates has increased rapidly in recent years. In this study, high-level carbapenem resistance and enhanced CAZ/AVI resistance were observed in two hypervirulent carbapenem-resistant *K. pneumoniae* isolates, KP1878 and KP3034, following prolonged carbapenem use. Virulence phenotypes were confirmed using the string test and a *Galleria mellonella* larvae infection model. Real-time quantitative PCR revealed that the relative expression of *bla*_KPC−2_ in KP1878 and KP3034 was 2.4-fold and 11.6-fold higher, respectively, than that in the CAZ/AVI-susceptible KPC-Kp strain KP1880. Whole-genome sequencing showed that the *bla*_KPC−2_ gene resided within an identical 5,692-bp Δ*klcA*-*korC*-ΔIS*Kpn6*-*bla*_KPC−2_-IS*Kpn8*-Δ*tnpR-*IS*26* tandem repeat, which was replicated twice and four times in plasmids pKPC1878 and pKPC3034, respectively. Compared with KP1880, the β-lactamase hydrolysis activities of crude cell lysates derived from KP1878 and KP3034 were significantly higher in their ability to hydrolyze meropenem, ceftazidime, and nitrocefin. S1-nuclease-digested pulsed-field gel electrophoresis, along with Southern blot and restriction fragment length polymorphism fingerprinting, identified plasmid profiles but revealed one or more 5.6-kilobase variations in the regions hybridized with the KPC-specific probe. Further comparative genomic analysis suggested that a potential homologous recombination event occurred between the *bla*_KPC−2_-carrying plasmid and the pLVPK-like virulence plasmid of KP3034, leading to the generation of a cointegrated plasmid that combined both virulence and CAZ/AVI resistance.

## 1 Introduction

With the widespread use of broad-spectrum antibiotics in clinical practice, multidrug-resistant (MDR) *Enterobacteriaceae* pose a serious public health challenge. *Klebsiella pneumoniae*, a member of the *Enterobacteriaceae* family, is now well recognized as a prominent opportunistic pathogen responsible for diverse community-acquired and nosocomial infections (Navon-Venezia et al., 2017). Nevertheless, the emergence of carbapenem-resistant *K. pneumoniae* (CRKP) has undermined the efficacy of carbapenems, previously regarded as potent therapeutic options for extended-spectrum β-lactamase (ESBL)- or AmpC-producing *Enterobacteriaceae* in clinical settings. Because of their widespread antimicrobial resistance and hypervirulence, CRKP colonization frequently correlates with compromised immune systems, unfavorable prognoses, and high morbidity and mortality (Nelson et al., 2017; Paczosa and Mecsas, 2016). In addition to “classical” carbapenem-resistant *K. pneumoniae* (cKp), hypervirulent carbapenem-resistant *K. pneumoniae* (Hv-CRKP) and carbapenem-resistant hypervirulent *K. pneumoniae* (CR-HvKP) combine both hypervirulence and carbapenem resistance, and have caused fatal outbreaks in hospitals in recent years (Gu et al., 2018). There is thus an urgent need for safe and effective strategies to control and prevent *K. pneumoniae* infections.

In response to the limited therapeutic options for severe and complex infections caused by Hv-CRKP and CR-HvKP isolates, ceftazidime/avibactam (CAZ/AVI)—a combination of a broad-spectrum cephalosporin and a non-β-lactam diazabicyclooctane (DBO) inhibitor—received approval from the Food and Drug Administration (FDA) in 2015 as a last-resort antimicrobial agent against multidrug-resistant Gram-negative bacilli (Van Duin and Bonomo, 2016; Zhanel et al., 2013). It is effective against carbapenem-resistant *Enterobacteriaceae* (CRE), which can lead to complicated infections such as intra-abdominal infections, urinary tract infections (UTIs), pyelonephritis, hospital-acquired pneumonia, and ventilator-associated pneumonia (Papp-Wallace et al., 2020). Avibactam acts by forming a reversible covalent bond with β-lactamases, effectively inhibiting extended-spectrum β-lactamases (ESBLs), Ambler class A serine carbapenemases (particularly KPC), AmpC β-lactamases, and OXA-48 carbapenemases, although it lacks activity against metallo-β-lactamases (MBLs) and some class D carbapenemases (Ehmann et al., 2012).

Since its discovery, CAZ/AVI resistance in KPC-Kp strains has been on the rise. Proposed mechanisms include mutations in the Ω-loop (Arg164 to Asp179) and hypervariable hotspots (Loop102–106, Loop238–243, Loop267–275) (Hobson et al., 2020, 2022), elevated *bla*_KPC_ gene copy numbers or plasmid acquisition (Coppi et al., 2020; Li et al., 2022), modifications in penicillin-binding proteins (PBPs) (Zhang et al., 2017), and alterations in outer membrane proteins accompanied by enhanced efflux activity (Contreras-Gómez et al., 2022; Shen et al., 2017). Despite numerous studies investigating the epidemiology of CAZ/AVI resistance in KPC-Kp isolates, the mechanism underlying *bla*_KPC−2_ copy number expansion and intrinsic CAZ/AVI resistance remains to be fully elucidated. In this work, we describe the unexpected emergence of CAZ/AVI resistance in two CRKP strains lacking prior exposure, quantify the copy number and relative expression of the *bla*_KPC−2_ gene, validate their β-lactam hydrolytic capacity, and elucidate how the *bla*_KPC−2_ copy number is increased in multidrug-resistant IncFII/IncR plasmids.

## 2 Materials and methods

### 2.1 Collection of clinical strains and antibiotic susceptibility testing

Two CAZ/AVI-non-susceptible *K. pneumoniae* strains, KP1878 and KP3034, were isolated from the residual specimens of ICU patients undergoing extended carbapenem therapy, out of 40 KPC-producing *K. pneumoniae* strains collected between 2015 and 2017 at a teaching hospital affiliated with Wenzhou Medical University (Liu et al., 2021). KP1880, a previously sequenced CAZ/AVI-susceptible strain that shared ~85% homology in PFGE patterns with KP1878 and KP3034 in our earlier research, was selected as a reference strain. Antibiotic susceptibility testing (AST) was performed using the VITEK 2 System (BioMérieux, Marcy l'Etoile, France) and further confirmed by the broth microdilution and agar dilution methods. Results were interpreted according to the Clinical and Laboratory Standards Institute (CLSI, 2022) guidelines and the Food and Drug Administration (FDA) breakpoints (for tigecycline and CAZ/AVI). *Escherichia coli* ATCC 25922 and *K. pneumoniae* ATCC 700603 served as quality control strains.

### 2.2 Genomic sequencing and bioinformatic analysis

Genomic DNA was extracted from clinical *K. pneumoniae* isolates using a bacterial genomic DNA extraction kit (Generay, Shanghai, China) and subjected to whole-genome sequencing (WGS) on both Illumina HiSeq 2500 and Oxford Nanopore platforms (Personalbio Technology, Shanghai, China). The raw sequencing data were initially assembled using Canu v2.1 (Koren et al., 2017), and the resulting draft genomes were subsequently polished with Illumina HiSeq reads via Pilon v1.23 (Walker et al., 2014). Gene annotation was carried out using the Prokka pipeline (Seemann, 2014). The presence of antimicrobial resistance (AMR) genes, virulence genes, plasmid profiles, and multilocus sequence types (MLST) was determined by aligning the assembled genomes against the ResFinder, VFDB, and PlasmidFinder databases in ABRicate v0.9.7 (https://github.com/tseemann/abricate) and Kleborate v2.2.0 (Lam et al., 2021). A plasmid homologous recombination evolution map was generated using Adobe Illustrator based on Basic Local Alignment Search Tool (BLAST) results. Nucleotide sequences of outer membrane porins were aligned with BLASTN, while polypeptide sequences were aligned using ClustalW and visualized via ESPript v3.0 (Gouet et al., 2003).

### 2.3 Comparative genomics analysis

The annotated virulence plasmids were compared with the hypervirulent plasmid pLVPK (NCBI GenBank Accession No. AY378100) using Gviewer (https://server.gview.ca/). The KPC-producing plasmids were aligned with previously sequenced plasmids pKPC2_200001 (Accession No. CP031720) and pSH9-KPC (Accession No. MH255827), both known to harbor multiple *bla*_KPC−2_ copies. Pairwise comparisons were then visualized using Easyfig v2.2.3.

### 2.4 Virulence assay

The string test for evaluating the hypermucoviscous phenotype was performed on blood agar as previously described (Shon et al., 2013). For the *Galleria mellonella* larvae infection model, overnight cultures of the Hv-CRKP isolates were adjusted to 0.5 McFarland in PBS, then diluted to a final density of 10^6^ CFU/mL. Larvae of *G. mellonella* (16 per group) were infected with clinical isolates and treated contralaterally with ceftazidime and avibactam at a fixed 4:1 ratio, as described elsewhere (Göttig et al., 2019). Larvae injected with 10 μL of PBS alone served as a negative control. Following incubation at 37°C, larval survival was recorded every 12 h, and the virulence assay was performed in triplicate. Mortality rates were evaluated using the log-rank test.

### 2.5 Plasmid manipulation

Plasmids from the Hv-CRKP isolates were separated by S1-nuclease-digested pulsed-field gel electrophoresis (S1-PFGE) and extracted using a Qiagen Plasmid Midi Kit (Qiagen, Germany). The resulting plasmid DNA was introduced into *E. coli* DH5α by electroporation, and transformants were selected on Mueller-Hinton (MH) agar plates containing 100 mg/L ampicillin. Putative transformants were verified via PCR amplification of the *bla*_KPC−2_ gene. KPC-producing plasmids were then isolated from the confirmed transformants and subjected to restriction digestion with *Nhe*I and *Spe*I. Electrophoresis was performed in a 1% agarose gel with 0.5× TBE buffer using the CHEF Mapper System (Bio-Rad, USA) at 14°C for 10 h. Southern blot hybridization was carried out with a digoxigenin-labeled *bla*_KPC−2_ probe, following the manufacturer's instructions for the DIG-High Prime DNA Labeling and Detection Starter Kit I (Roche, Germany).

### 2.6 Isolation of outer membrane proteins and detection of β-lactamase hydrolysis activity

Outer membrane proteins (OMPs) from *K. pneumoniae* isolates were isolated by sonicating bacterial cultures grown in MH broth, followed by the solubilization of total membrane fractions with 2% (wt/vol) sodium lauryl sarcosylate and ultracentrifugation (García-Fernández et al., 2010). The inner membrane was selectively solubilized while the outer membrane remained intact, as previously described (Filip et al., 1973). *K. pneumoniae* ATCC 13883 served as a control for protein profile comparisons. Proteins were separated by SDS-PAGE and stained with Coomassie blue. β-Lactamase hydrolysis activity was monitored in crude supernatants from sonicated cells of three independent cultures of each strain, using 100 μM meropenem, nitrocefin, or ceftazidime as substrates, in the absence or presence of 4 mg/L avibactam. The molar absorptivity coefficient (M^−1^cm^−1^) and wavelength (nm) for measuring substrate hydrolysis were 8,660 M^−1^cm^−1^ at 260 nm for ceftazidime, 10,400 M^−1^cm^−1^ at 299 nm for meropenem, and 17,420 M^−1^cm^−1^ at 486 nm for nitrocefin, respectively (Marumo et al., 1996). One unit of enzyme activity was defined as the amount of enzyme required to hydrolyze 1 nmol of substrate per minute per milligram of protein.

### 2.7 Conjugation assay

Conjugation experiments were performed as previously described (Liu et al., 2021). Briefly, donor *K. pneumoniae* strains and rifampicin-resistant *E. coli* EC600 were mixed at a 1:1 ratio on LB agar and incubated at 37°C for 12 h. Conjugants were selected on MacConkey agar supplemented with rifampicin (1,024 mg/L) and meropenem (2 mg/L). Conjugation efficiency was calculated as the number of conjugants per donor cell.

### 2.8 Quantification of *bla*_kpc−2_ transcript expression and copy number prediction

Absolute quantification of the *bla*_KPC−2_ copy number and relative expression levels was performed using real-time quantitative PCR (qPCR) and quantitative reverse-transcription PCR (qRT-PCR), as previously reported (Kitchel et al., 2010; Nelson et al., 2017). Primer sequences are listed in Supplementary Table 1. Purified amplicons of a 285-bp *bla*_KPC−2_ fragment and a 147-bp *pgi* fragment were individually ligated into pEASY-Blunt vectors (TransGen Biotech, Beijing, China) and transformed into *Escherichia coli* DH5α, serving as plasmid standards for quantification. Standard curves for both the target (*bla*_KPC−2_) and the internal control (*pgi*) were generated using 10-fold serial dilutions of the purified plasmid extracts at known concentrations (Nelson et al., 2017). Each quantification was performed in triplicate. The copy number of *bla*_KPC−2_ was further estimated using the CCNE calculator (https://github.com/biojiang/ccne) and assessed by comparing read-depth coverage to that of *pgi* (Jiang et al., 2022).

### 2.9 Statistical analysis

All statistical analyses were performed using SPSS 23.0 and GraphPad Prism 9.3.1. Comparisons of β-lactamase hydrolysis activity and qPCR results were conducted via one-way ANOVA. Differences with ^*^P < 0.05, ^**^P < 0.01, or ^***^P < 0.001 were considered statistically significant.

## 3 Results

### 3.1 Clinical and antimicrobial profiles of KPC-Kp isolates

High-level carbapenem resistance was observed in two KPC-Kp strains (KP1878 and KP3034) isolated from patients who developed nosocomial infections in the ICU. Antimicrobial susceptibility testing revealed that both strains were non-susceptible to CAZ/AVI and resistant to all β-lactams and carbapenems tested, yet remained susceptible to trimethoprim-sulfamethoxazole, tigecycline, and polymyxin B. All *E. coli* transformants harboring *bla*_KPC−2_-bearing plasmids exhibited low-level resistance to carbapenems. Retrospective review of clinical data showed that both patients had received cephalosporins and carbapenems prior to pathogen isolation, but had not been treated with CAZ/AVI-based regimens. The prescribed antimicrobial therapies and clinical characteristics of these patients are summarized in Table 1.

**Table 1:** Characteristics of carbapenemase-producing *Klebsiella pneumoniae* isolates and transformants with *bla*_KPC−2_-carrying plasmids.

**Patients**	**Isolates**	**Source**	**Diagnosis**	**Prescribed antimicrobials**	**MIC (mg/L)**									**Length of stay (Days)**	**Clinical outcomes**
					**MEM**	**IPM**	**ETP**	**CAZ**	**CAZ/AVI**	**GEN**	**SXT**	**TGC**	**POL**		
1	KP1878	Drainage liquid	Acute pancreatitis	IPM, PAN, MEM	256	64	512	64	8	1024	8	1	2	44	Discharged
1	DH5α (pKPC1878)	Transformant^*^	-	-	2	8	8	8	0.125	512	≤0.5	≤0.5	≤0.5	-	-
2	KP1880	Wound exudation	Pressure ulcer	MEM, CSL, PAN	128	32	256	64	2	1,024	8	2	2	54	Discharged
2	DH5α (pKPC1880)	Transformant^*^	-	-	2	4	4	16	0.5	1,024	≤0.5	≤0.5	≤0.5	-	-
3	KP3034	Urine	Severe sepsis	CXM, TZP, MEM	1024	512	1024	256	16	≤0.5	8	1	1	134	Died
3	DH5α (pKPC3034)	Transformant^*^	-	-	2	4	8	8	0.25	≤0.5	≤0.5	≤0.5	≤0.5	-	-

IPM, imipenem; PAN, panipenem; MEM, meropenem; CSL, cefoperazone/sulbactam; CXM, cefuroxime; TZP, piperacillin/tazobactam; ETP, ertapenem; CAZ, ceftazidime; CAZ/AVI, ceftazidime/avibactam; GEN, gentamicin; SXT, trimethoprim-sulfamethoxazole; TGC, tigecycline; PMB, polymyxin B.

^*^E. coli transformants with *bla*_KPC−2_-carrying plasmids deprived from wild-type CRKP strains.

### 3.2 Genomic analysis of AMR and virulence determinants, and replicon types of plasmids in Hv-CRKP strains

The genetic characteristics of plasmids associated with antimicrobial resistance (AMR) and virulence in KPC-Kp strains are summarized in Table 2. All strains belonged to sequence type ST11 and serotype KL47 (ST11-KL47 clones), and carried IncH1B virulence plasmids, IncFII/IncR *bla*_KPC−2_-bearing MDR plasmids, as well as ColRNAI plasmids. All strains harbored identical resistance genes for β-lactams, fosfomycin, and chloramphenicol; however, KP3034 lacked aminoglycoside resistance genes (e.g., *rmtB*), rendering it susceptible to aminoglycosides. The *bla*_KPC−2_ gene was located on the MDR plasmids and served as the sole determinant of carbapenem resistance. Virulence factors, including iron acquisition systems salmochelin (*iro*) and aerobactin (*iuc, iut*) synthesis loci, as well as the hypermucoviscosity regulators *rmpA/rmpA2*, were identified in all Hv-CRKP strains tested. Notably, the KPC-carrying plasmid pKPC3034 combined both virulence and carbapenem resistance determinants.

**Table 2:** Genomic characteristics of hypervirulent carbapenem-resistant *Klebsiella pneumoniae* strains.

**Strains**	**Genome**	**Size (bp)**	**Serotypes/Plasmid replicons**	**Antimicrobial resistance and virulence determinants**
KP1878	chromosome	5,428,703	KL47	*bla*_SHV − 11_
	pVIR1878	217,255	IncH1B	*iucABCD, iutA, rmpA2*
	pKPC1878	174,654	IncFII/IncR	*rmtB, fosA3, catII*, *bla*_TEM − 1_, *bla*_CTX − M−65_, *bla*_KPC−2_
	pCOL1878	6,796	ColRNAI	ND
KP1880	chromosome	5,439,139	KL47	*bla*_SHV − 11_
	pVIR1880	227,278	IncH1B	*iucABCD, iutA, iroD, iroN, rmpA, rmpA2*
	pKPC1880	168,960	IncFII	*rmtB, fosA3, catII*, *bla*_TEM − 1_, *bla*_CTX − M−65_, *bla*_KPC−2_
	pMDR1880	124,930	IncFII_K_	*aac(3)-IId, aac(6′)-Ib, aadA16, aph3-Ia, strA, strB, mphA, floR, arr-3, sul1, sul2, tet(A), dfrA27*
	pCOL1880	6,796	ColRNAI	ND
KP3034	chromosome	5,434,979	KL47	*bla*_SHV − 11_
	pVIR3034	214,285	IncH1B	*catII, iucABCD, iutA, rmpA2*
	pKPC3034	200,740	IncFII/IncR	*fosA3*, *bla*_TEM − 1_, *bla*_CTX − M−65_, *bla*_KPC−2_, *iroD, iroN, rmpA*
	pCOL3034	6,796	ColRNAI	ND

ND: not detected.

### 3.3 Increased expression and copy numbers of *bla*_kpc−2_ in KPC-Kp isolates

**Figure 1:**
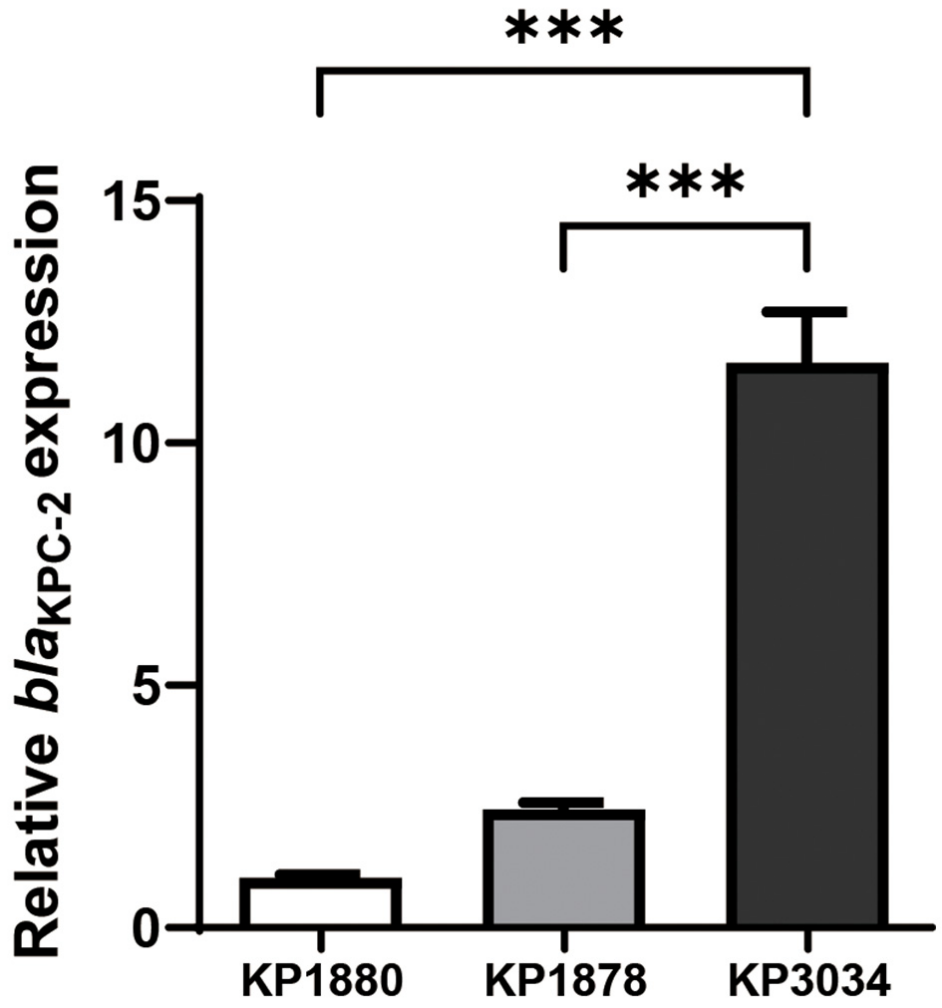
Relative expression of the *bla*_KPC−2_ gene in clinical *Klebsiella pneumoniae* strains. Statistical analysis was performed using one-way ANOVA. Differences with ***P < 0.001 were considered statistically significant.

Quantitative PCR was used to evaluate the ratio of *bla*_KPC−2_ copy number relative to that of the housekeeping gene. A comparison of standard Illumina sequencing reads and subsequent WGS data confirmed that *bla*_KPC−2_ copy numbers doubled in pKPC1878 and quadrupled in pKPC3034 (Supplementary Table 2). In addition to their high-level carbapenem resistance, KP1878 and KP3034 displayed CAZ/AVI non-susceptibility, with minimum inhibitory concentrations (MICs) of 8/4 mg/L and 16/4 mg/L, respectively (Table 1). RT-qPCR analyses indicated that the relative expression of *bla*_KPC−2_ increased by 11.6-fold in KP3034 (*P* < 0.05) and 2.4-fold in KP1878 (*P* = 0.06), compared with the reference KPC-Kp strain KP1880 (Figure 1).

### 3.4 Comparative genomic analysis of plasmids

**Figure 2:**
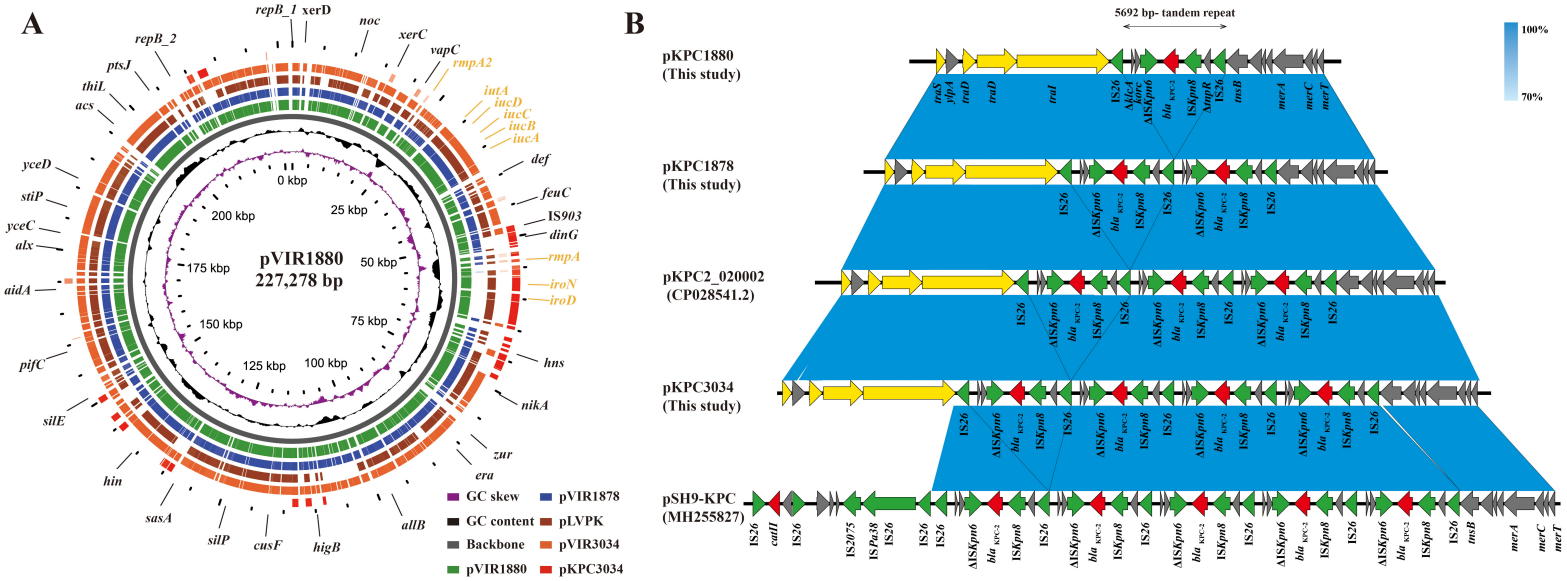
Comparative analysis of hypervirulent plasmids and alignment of *bla*_KPC−2_ regions in KPC-producing plasmids. **(A)** Comparation of virulence-encoding plasmids with the hypervirulent plasmid pLVPK (GeneBank Accession No. NC_005249). From center to periphery: pVIR1880, pVIR1878, pLVPK, pVIR3034 and pKPC3034. Virulence genes are shown in earthen yellow. **(B)** Linear comparison of the *bla*_KPC−2_-encoding tandem repeats and homologous regions in the NCBI Nucleotide database. Genes with distinct functions are colored as follows: red, antibiotic resistance; green, mobile genetic elements; yellow, transfer conjugation.

A comparative genomic analysis of virulence plasmids, including the virulence gene–harboring plasmid pKPC3034 and the hypervirulent plasmid pLVPK (GenBank Accession No. NC_005249), revealed that the virulence plasmids in Hv-CRKP share remarkably similar genetic structures and sizes (Figure 2A). The virulence region of pKPC3034 shared strong homology with pVIR1880, while pVIR3034 exhibited 99.9% identity and 96% coverage with pVIR1880 and pVIR1878, respectively. This region could be complemented to form a pLVPK-like plasmid by acquiring the homologous *iroD-iroN-rmpA* region from pKPC3034.

A linear comparison of *bla*_KPC−2_-bearing regions against publicly available sequences in the NCBI GenBank database demonstrated close similarity to two previously reported plasmids: the IncFII/IncR plasmid pKPC2_200001, which carries three copies of *bla*_KPC−2_, and the IncR plasmid pSH9-KPC, which carries five copies of *bla*_KPC−2_ (Figure 2B). In these *bla*_KPC−2_-bearing plasmids, flanking IS*26* elements and the intervening Δ*klcA*-*korC*-Δ*ISKpn6*-*bla*_KPC−2_-*ISKpn8*-Δ*tnpR* region form a non-Tn4401 element (NTE_KPC_-Id) composite transposon, with a total length of 6,409 bp. The core *bla*_KPC−2_ region adjacent to IS*26* generates a 5,692-bp tandem unit (Δ*klcA*-*korC*-Δ*ISKpn6*-*bla*_KPC−2_-*ISKpn8*-Δ*tnpR*-IS*26*), duplicated twice and four times in pKPC1878 and pKPC3034, respectively. Notably, while plasmids pKPC1880 and pKPC1878 successfully transferred via conjugation to *E. coli* EC600 (conjugation frequencies of 4.35 × 10^−5^ and 2.04 × 10^−5^, respectively), pKPC3034 failed to conjugate, likely owing to its large size (~200 kb) or incompatibility with the recipient host.

### 3.5 Plasmid characterization and analysis

**Figure 3:**
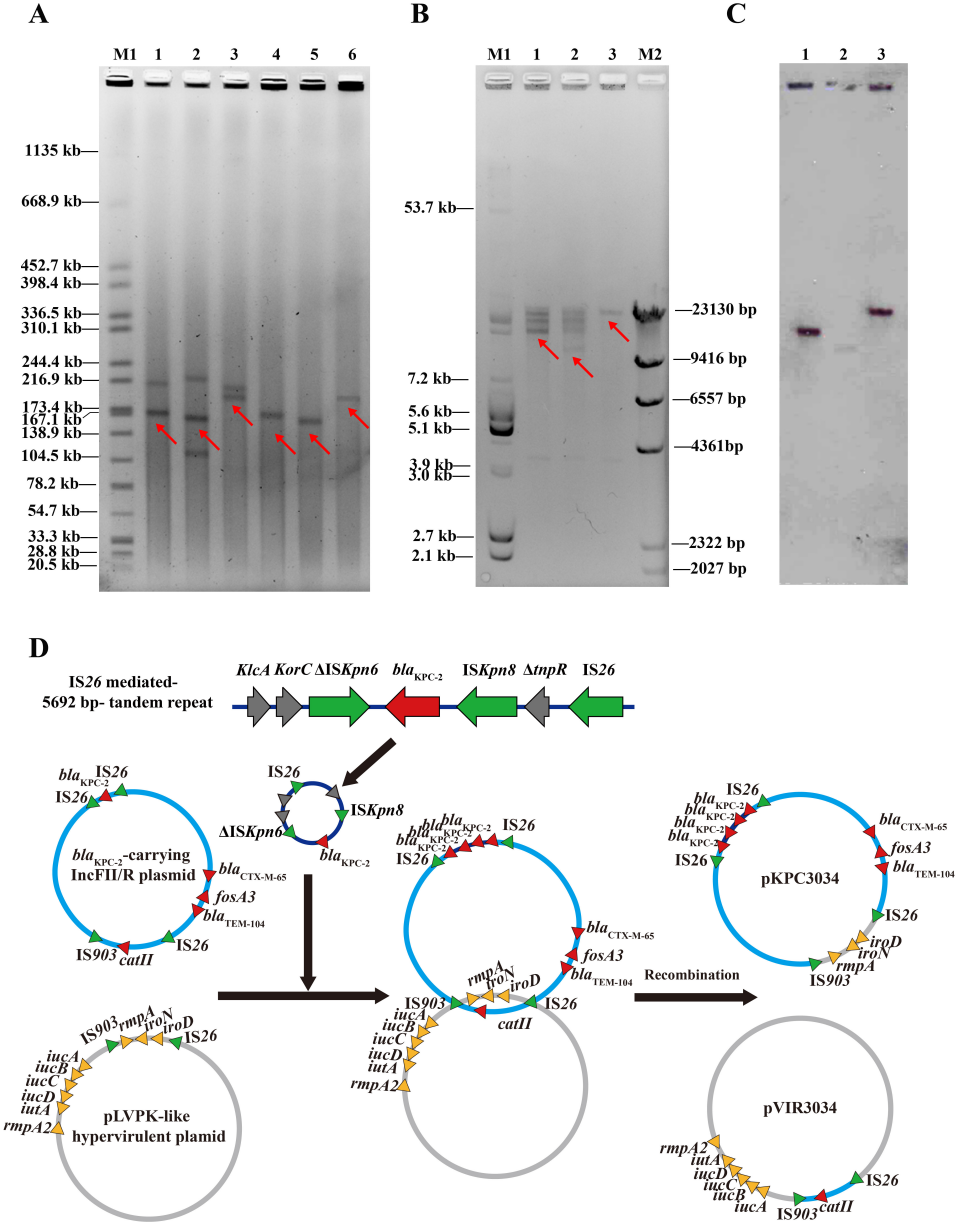
Identification of plasmids with increased *bla*_KPC−2_ gene copy numbers. **(A)** S1-PFGE analysis of wild-type strains and transformants. Lane 1: KP1878; Lane 2: KP1880; Lane 3: KP3034; Lane 4: DH5α (pKPC1878); Lane 5: DH5α (pKPC1880); Lane 6: DH5α (pKPC3034). **(B)** Gel electrophoresis of *Nhe*I- and *Spe*I-digested KPC-producing plasmids. **(C)** Southern blot hybridization with a *bla*_KPC_-specific probe. M1: *E. coil* V517 plasmids, M2: λ-*Hind*III DNA ladder; Lane 1: DH5α (pKPC1878); Lane 2: DH5α (pKPC1880); Lane 3: DH5α (pKPC3034). **(D)** Schematic diagram illustrating plasmid evolution and recombination in *K. pneumoniae* strain KP3034. pKPC3034 acquired additional *bla*_KPC−2_ copies via IS*26*-mediated tandem replication and exchanged homologous region with pVIR3034.

S1-PFGE results for representative clinical isolates and transformants showed that the *bla*_KPC−2_ gene resided on MDR plasmids ranging from 160 to 200 kb (Figure 3A). Similar patterns of RFLP restriction bands were observed in both RFLP analysis and Southern blot hybridization using plasmid DNA extracted from transformants (Figures 3B, C). The *bla*_KPC−2_ probe hybridized to a region between 10 and 30 kb, aligning with the *Nhe*I and *Spe*I restriction fragment profiles (Supplementary Table 3). Notably, these profiles differed from one another by approximately 5,692 bp. Plasmids pKPC1880 and pKPC1878 yielded identical fragments except at the *bla*_KPC−2_ hybridization sites (11.6 and 17.9 kb, respectively). By contrast, in plasmid pKPC3034, the hybridized fragment was around 29 kb (Figure 3C). These findings support the presence of *bla*_KPC−2_-bearing tandem repeats in pKPC1878 and pKPC3034.

To elucidate how the resistance–virulence cointegrated plasmid pKPC3034 arose, we constructed a schematic diagram depicting the proposed recombination process (Figure 3D). First, the 5,692-bp Δ*klcA*-*korC*-Δ*ISKpn6*-*bla*_KPC−2_-*ISKpn8*-Δ*tnpR*-IS*26* tandem repeat functioned as a composite transposon in an IS*26*-mediated homologous recombination event, subsequently undergoing quadruple replication and fusion with an IncFII/IncR pKPC1880-like multidrug-resistant plasmid. The *iroD-iroN-rmpA* region in the virulent plasmid pVIR3034 was deleted, replaced by an IS*26*-*catII*-IS*26* module encoding the chloramphenicol resistance gene *catII*. Further sequence analysis revealed that the *iroD-iroN-rmpA* region in pKPC3034 was homologous to that in pVIR1880, whereas the *catII* locus in pVIR3034 was homologous to pKPC1880. In addition, both IS*26* and IS*903* flanked these homologous segments across the aforementioned plasmids. The pKPC1880-like plasmid pKPC3034 and the pLVPK-like plasmid pVIR3034 then exchanged homologous regions through IS*26*- and IS*903*-based composite transposition. As a result, pKPC3034 acquired virulence genes from pVIR3034, while pVIR3034 gained additional chloramphenicol resistance.

### 3.6 β-lactamase hydrolysis activity and outer membrane protein production

**Figure 4:**
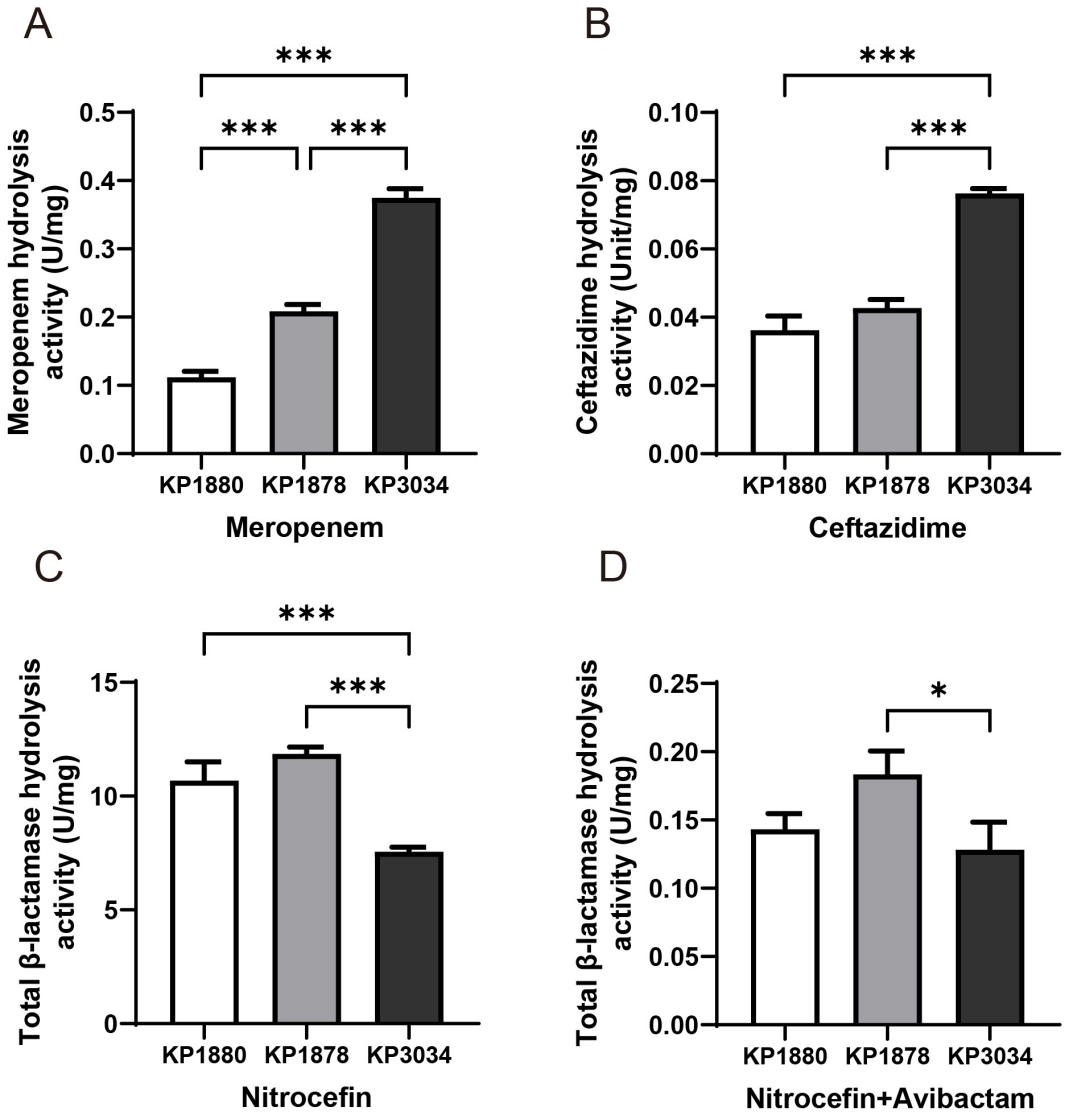
β-Lactamase hydrolytic activity in crude cell lysates of clinical *Klebsiella pneumoniae* strains (U/mg). **(A)** Ceftazidime hydrolysis activity. **(B)** Meropenem hydrolysis activity. **(C)** Total β-lactamase activity using nitrocefin as a substrate. **(D)** Total β-lactamase activity in the presence of 4 mg/L avibactam. Statistical analysis was performed using one-way ANOVA. Differences with *P < 0.05, and ***P < 0.001 were considered statistically significant.

Among the selected strains, KP3034 exhibited the highest hydrolytic activity toward meropenem and ceftazidime (Figures 4A, B), aligning with its enhanced ceftazidime and carbapenem resistance. By contrast, KP1880 and KP1878 showed no significant differences in their capacity to hydrolyze ceftazidime or nitrocefin (Figures 4B, C). The addition of 4 mg/L avibactam significantly reduced total β-lactamase activity by nearly 100-fold (Figures 4C, D). However, when avibactam was added to ceftazidime, the kinetic parameters of enzymatic hydrolysis were too low to be measured (data not shown).

Regarding outer membrane porins, genetic analysis revealed a frameshift mutation at amino acid 29 in outer membrane protein OmpK35 (c.85_86delA), resulting in a truncated 38-amino-acid polypeptide (Supplementary Figure 1). A glycine-aspartate (GD) insertion at positions 134 and 135 in the extracellular loop 3 region of OmpK36 was also identified. On SDS-PAGE, the ~37 kDa band likely corresponds to the altered OmpK36, whereas OmpK35 was absent in all tested strains (Supplementary Figure 2).

### 3.7 Virulence assay

**Figure 5:**
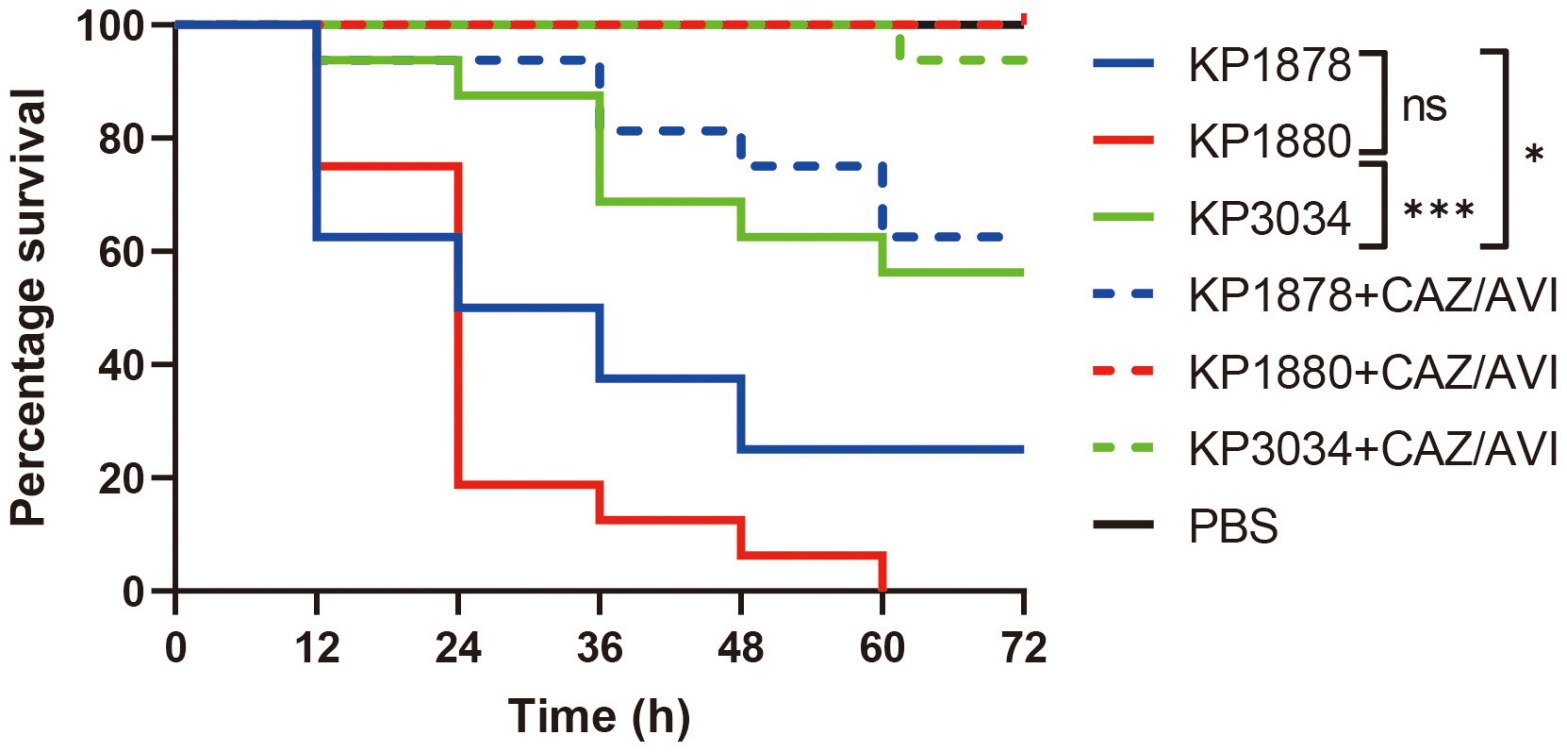
Survival curves in the *Galleria mellonella* larvae infection model. Differences between survival curves were evaluated using the log-rank test. *P < 0.05 and ***P < 0.001 were considered statistically significant.

All strains exhibited hypermucoviscosity (positive string test) and carried virulence-associated loci. However, genomic analysis revealed varying virulence gene compositions: KP1880 retained a full complement of virulence determinants (*iucABCD-iutA, iroD-iroN, rmpA/rmpA2*), whereas KP1878 lacked *iroD-iroN*, and KP3034 lost *iroD-iroN-rmpA* in its virulence plasmid as a result of IS*26*-mediated recombination (Table 2). These genetic differences correlated with virulence phenotypes in the *Galleria mellonella* model (Figure 5). KP1880 caused 100% mortality within 60 h, reflecting its intact virulence arsenal. KP1878, which lacked salmochelin biosynthesis (*iroD-iroN*), showed reduced lethality (75% mortality at 72 h). KP3034, harboring a disrupted *iroD-iroN-rmpA* region and a cointegrated plasmid, exhibited the lowest virulence (44% mortality), underscoring the importance of these loci in systemic infection.

Administration of CAZ/AVI significantly improved larval survival and was most effective in rescuing strains that were CAZ/AVI-susceptible (dotted lines in Figure 5, *P* < 0.05). However, CAZ/AVI therapy was insufficient to enhance survival in infections caused by KP1878 compared with the other two strains.

## 4 Discussion

ST11 type *K. pneumoniae* strains are frequently associated with hypervirulence and multidrug resistance, and the emergence of Hv-CRKP has led to severe outbreaks in hospitals (Lee et al., 2017; Li et al., 2021). Such strains combine elevated virulence and extensive drug resistance, resulting in high morbidity, mortality, and limited therapeutic options in settings like the ICU. In our study, patients developed severe nosocomial infections with ST11 Hv-CRKP following prolonged β-lactam and carbapenem usage. Whole-genome sequencing revealed that these isolates carried two or four copies of the *bla*_KPC−2_ gene on IncFII/IncR plasmids, each copy embedded within a 5,692-bp Δ*klcA*-*korC*-IS*Kpn6*-*bla*_KPC−2_-IS*Kpn8*-*tnpR*-IS*26* tandem repeat. As vehicles for horizontal gene transfer, the mobilization of such translocatable units contributes to the increased copy number of *bla*_KPC−2_ in carbapenem-resistant plasmids and significantly contributed to CAZ/AVI non-susceptibility (Li et al., 2022). In our earlier work, the *bla*_KPC−2_-bearing plasmids pKPC1880 and pKPC1878 were conjugation-competent, suggesting that they could disseminate accumulated carbapenem resistance to other members of the *Enterobacteriaceae* hosts (Liu et al., 2021).

From a clinical perspective, the co-occurrence of hypervirulence and multiple *bla*_KPC−2_ copies in one strain underscores an alarming trend of “convergent evolution”, where virulence and antimicrobial resistance accumulate together (Arcari and Carattoli, 2023). Our *Galleria mellonella* assays confirmed that these isolates retained hypermucoviscous phenotypes and caused high mortality *in vivo*, while still displaying expanded resistance to carbapenems and CAZ/AVI. In particular, strain KP3034 harbored a hybrid IncFII/IncR plasmid comprising multiple copies of *bla*_KPC−2_ along with segments of the virulence genes (*iroD*-*iroN*-*rmpA*). Notably, KP3034 also exhibited attenuated virulence linked to partial deletions in the native virulence plasmid (pVIR3034). Although the cointegrated plasmid (pKPC3034) reacquired *iroD*-*iroN*-*rmpA* from a pLVPK-like hypervirulent plasmid via recombination, potential transcriptional interference or suboptimal expression in this hybrid context may have led to reduced pathogenicity. Such plasmid rearrangements highlight the delicate balance between selective pressures favoring carbapenem resistance and the need for intact virulence determinants to maintain hypervirulent phenotypes (Tian et al., 2022; Yang et al., 2022b).

Our findings also underscore the broader importance of tracking carbapenem resistance and virulence plasmids in clinical *K. pneumoniae* isolates (Yang et al., 2021; Chen et al., 2020). The simultaneous emergence of hypervirulence and extensive resistance raises the risk of therapeutic failures and demands heightened infection control measures (Yang et al., 2022a). By combining phenotypic, genomic, and epidemiological analyses, we provide molecular level evidence that IS*26* and IS*903* facilitate plasmid recombination and, consequently, accelerate the fusion of antimicrobial resistance and virulence traits (Park et al., 2017; Hu et al., 2022; Yang et al., 2022b). Furthermore, we have shown that high copy *bla*_KPC−2_ can markedly elevate CAZ/AVI resistance, reinforcing the importance of plasmid surveillance (including copy-number assessments) in guiding early diagnostic and therapeutic strategies.

In conclusion, our study illuminates that ST11 Hv-CRKP can evolve through IS-mediated recombination events to generate resistance-virulence cointegrated plasmids that simultaneously harbor CAZ/AVI resistance and hypervirulent traits. The tandem replication of Δ*klcA*-*korC*-ΔIS*Kpn6*-*bla*_KPC−2_-IS*Kpn8*-Δ*tnpR*-IS*26* units led to increased *bla*_KPC−2_ copy numbers, accounting for the emergence of CAZ/AVI resistance in KPC-Kp strains. These findings underscore the urgent need for comprehensive surveillance, encompassing both microbiological and genomic approaches, to optimize treatment strategies and prevent the further spread of these high-risk clones.

## Data Availability

All primers used in this study are listed in Supplementary Table 1. Genome sequence data have been deposited at NCBI under BioProject No. PRJNA1062154.
