# Supplementary material for: Emergence and characterization of IncFII/IncR plasmids with multiple 5,692 bp- blaKPC−2-bearing tandem repeats in ceftazidime/avibactam non-susceptible Klebsiella pneumoniae strains
Source: Front Microbiol. 2025 Apr 3;16:1534631. doi: 10.3389/fmicb.2025.1534631 (PMC12003348; doi:10.3389/fmicb.2025.1534631)
Supplement: Supplementary file 1 [file Table_1.docx]

Table S1. Primers Used in This Study.

| Primer name | Sequence (5′ to 3′) | Size (bp) | Purpose |
| --- | --- | --- | --- |
| qRT-16sRNA-F | AATGCCACGGTGAATACG | 153 | Quantity PCR |
| qRT-16sRNA-R | CTACGGTTACCTTGTTACGA |  |  |
| qRT-KPC-F | AATTGGCGGCGGCGTTATCA | 285 | Quantity PCR/Cloning |
| qRT-KPC-R | GGCGGCTCCATCGGTGTGTA |  |  |
| qRT-PGI-F | TTCATCGCTCCGGCTATCAC | 148 | Quantity PCR/Cloning |
| qRT-PGI-R | CCGGGTCTTTACCCTGATCG |  |  |
| KPC-DIG-probe* | TGATAACGCCGCCGCCAATTTGT | 23 | Hybridization |

*Digoxin-labeled oligonucleotide.
