# Supplementary material for: Emergence and characterization of IncFII/IncR plasmids with multiple 5,692 bp- blaKPC−2-bearing tandem repeats in ceftazidime/avibactam non-susceptible Klebsiella pneumoniae strains
Source: Front Microbiol. 2025 Apr 3;16:1534631. doi: 10.3389/fmicb.2025.1534631 (PMC12003348; doi:10.3389/fmicb.2025.1534631)
Supplement: Supplementary file 2 [file Table_2.docx]

Table S2. Different Methodologies for Quantifying *bla*_KPC-2_ Gene Copy Number.

| Methods | Copy numbers | | |
| --- | --- | --- | --- |
|  | KP1880 | KP1878 | KP3034 |
| CCNE program | 1.41 | 2.76 | 6.56 |
| Real-time quantity PCR | 1.09 ± 0.01 | 0.98 ± 0.04 | 2.10 ± 0.03 |
| Nanopore sequencing | 1 | 2 | 4 |
