# Supplementary material for: Emergence and characterization of IncFII/IncR plasmids with multiple 5,692 bp- blaKPC−2-bearing tandem repeats in ceftazidime/avibactam non-susceptible Klebsiella pneumoniae strains
Source: Front Microbiol. 2025 Apr 3;16:1534631. doi: 10.3389/fmicb.2025.1534631 (PMC12003348; doi:10.3389/fmicb.2025.1534631)
Supplement: Supplementary file 3 [file Table_3.docx]

Table S3. Restriction Profiles of KPC-Carrying Plasmids Digested with *Nhe*I and *Spe*I.

| Plasmid | Length（bp） | *Spe*Ⅰ sites | *Nhe*Ⅰ sites |
| --- | --- | --- | --- |
| pKPC1878 | 1867, 4224, 17037, 17623^*^, 25929, 33687, 34110, 40185 | 1 | 7 |
| pKPC1880 | 1867, 4224, 11931^*^, 17037, 25929, 33687, 34110, 40183 | 1 | 7 |
| pKPC3034 | 2742, 2708, 2988, 4094, 11558, 26061, 29007^*^, 36639, 40185, 44773 | 3 | 7 |

*Location of *bla*_KPC-2_*-*bearing tandem repeats.
