# Supplementary material for: Emergence and characterization of IncFII/IncR plasmids with multiple 5,692 bp- blaKPC−2-bearing tandem repeats in ceftazidime/avibactam non-susceptible Klebsiella pneumoniae strains
Source: Front Microbiol. 2025 Apr 3;16:1534631. doi: 10.3389/fmicb.2025.1534631 (PMC12003348; doi:10.3389/fmicb.2025.1534631)
Supplement: Supplementary file 4 [file Table_4.docx]

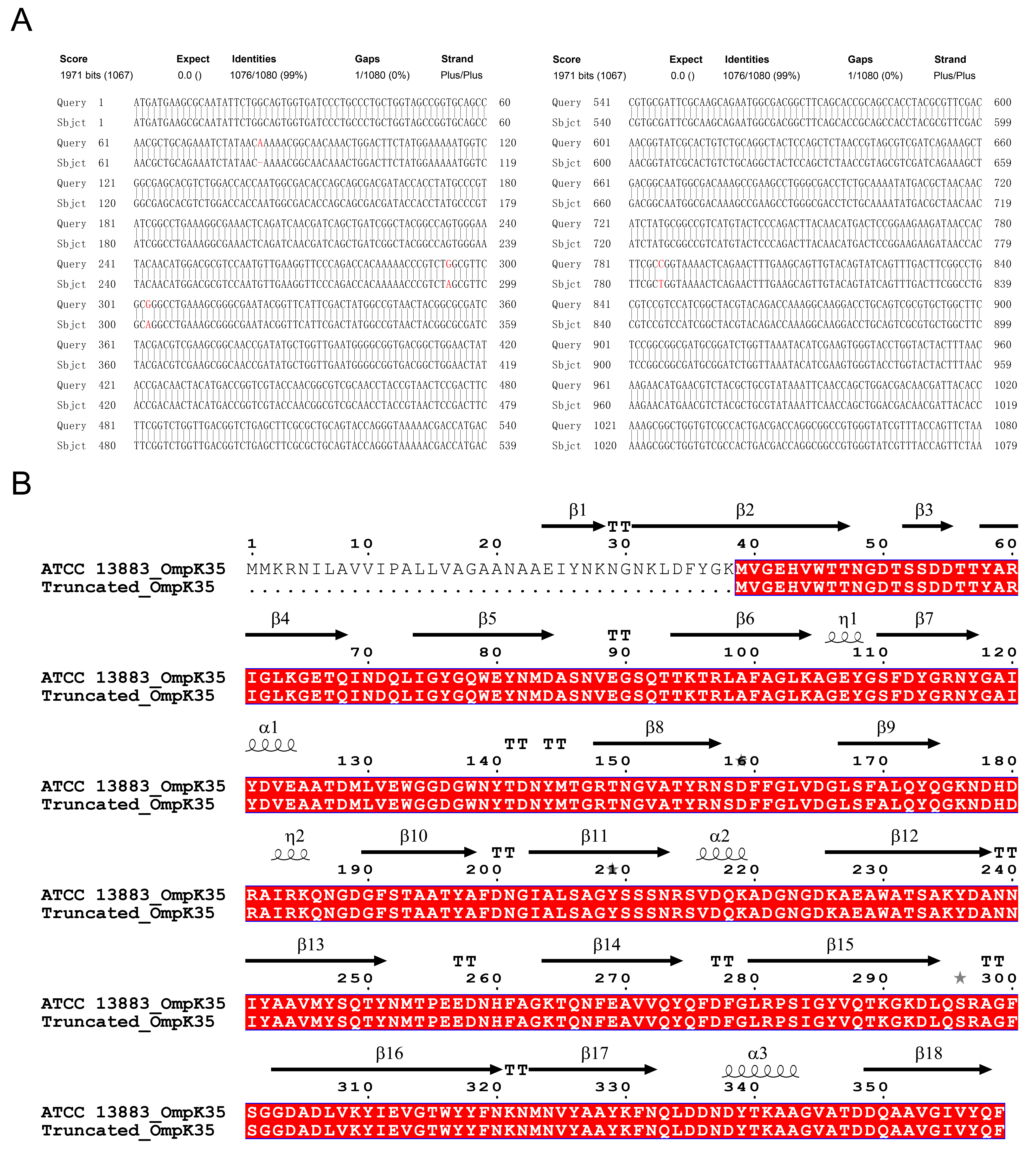


**Fig. S1. Alignment of Truncated OmpK35 Sequences from Clinical CRKP Strains with the Intact Outer Membrane Porin Sequence from ATCC 13883.** (A) Nucleotide alignment: The query represents the full-length OmpK35-encoding sequence from ATCC 13883, while the subject corresponds to the truncated OmpK35 sequences from clinical CRKP strains. Nucleotide variations are highlighted in red. (B) Amino acid (polypeptide) sequence alignment.
