# Supplementary material for: Emergence and characterization of IncFII/IncR plasmids with multiple 5,692 bp- blaKPC−2-bearing tandem repeats in ceftazidime/avibactam non-susceptible Klebsiella pneumoniae strains
Source: Front Microbiol. 2025 Apr 3;16:1534631. doi: 10.3389/fmicb.2025.1534631 (PMC12003348; doi:10.3389/fmicb.2025.1534631)
Supplement: Supplementary file 5 [file Table_5.docx]

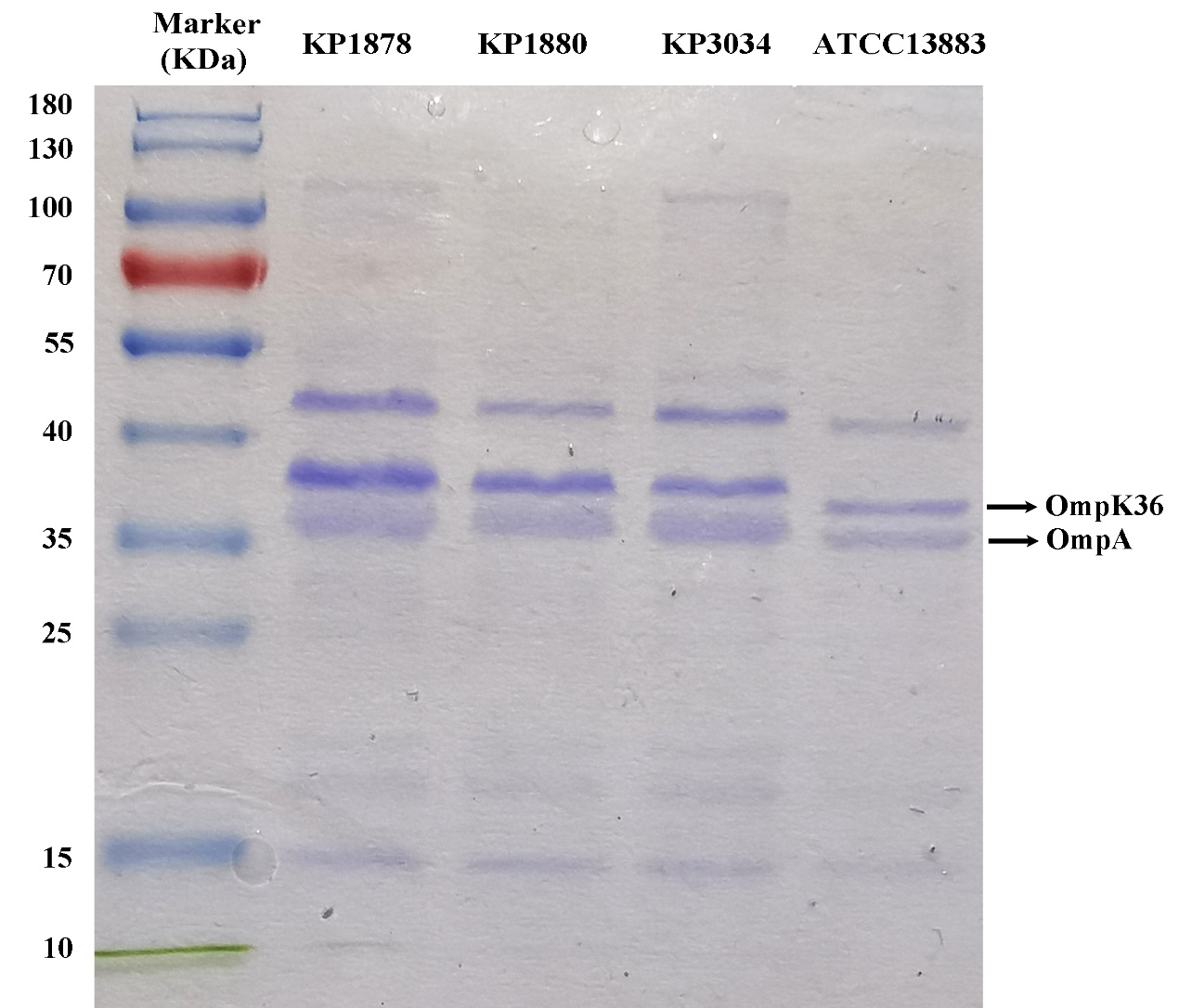


**Fig. S2**. Outer Membrane Protein Profiles of Clinical KPC-KP Isolates. Marker: protein markers. Lane 1-4: KP1878, KP1880, KP3034, *K. pneumoniae* ATCC 13883.
